# On Chronic Joint Diseases as Seen in Medical Out-Patients.—I

**Published:** 1899-03-11

**Authors:** Geo. Parker

**Affiliations:** Assistant Physician at the General Hospital, and Lecturer in the Medical School, University College, Bristol.


### Hospital Clinics and Medical Progress.

### ON CHRONIC JOINT DISEASES AS SEEN IN MEDICAL OUT-PATIENTS.—I.

By Geo. Parker, M.A., M.D., Assistant Physician at the General Hospital, and Lecturer in the Medical School, University College, Bristol.

Surgical joint affections are confessedly difficult to diagnose and to treat, but in those which belong to the physician we have special obstacles not found in all surgical cases. Local troubles are here either symptoms of general diseases or complicated by them. A joint injured by rheumatism may fail under the strain of daily work, which wears down the weakened tissue. Again, a joint may be picked out by gout for no known reason, or because it has previously been overworked or injured, much as in experimental septicæmia crowds of microbes are found gathered round an injured joint. Finally, a joint attacked by gout or gonorrhœa to-day may be attacked in after-life by acute rheumatism. The affections are not always inflammatory in origin; purely degenerative changes occur, but the main point to remember is that we have no hybrid diseases. The state of the joint may be the result of the action of two or three causes, but each of these causes is distinct, and produces its own peculiar effect. We have then to disentangle the effects of the local and general processes.

Nearly all diseases affecting the joints were once included under the term rheumatism, but one after another they have been separated from it and from each other. Gout, rheumatoid arthritis, tabes, and many septic disorders are now seen to be entirely distinct from it in origin and progress, while the limits of true rheumatism are year by year shown to be narrower, though even now many kinds of affections are referred o it which have probably no connection with it at all. Possibly we may find that no chronic joint diseases are exclusively due to rheumatism, though this disease complicates many such conditions at an early or later stage. Hence the man who treats all old joint troubles as rheumatic is quite as culpable as he would be were he to prescribe for diarrhœa without regard to any possible underlying kidney disease or typhoid fever. The temporary effect of treatment, again, is not a safe guide, for many symptoms, and particularly pain, may be lessened for a while by rest, applications of hot air or fomentations, and salicylates externally or internally. To do permanent good much more is needed, but permanent good can be often obtained if a correct diagnosis be made at an early stage. Now, in regard to diagnosis, not only may the appearance of a joint be deceptive, from the mixture of local and general causes, but the history of former illnesses is unreliable. Previous attacks of so-called rheumatism may mean anything. Rheumatic fever patients, by the way, are rather ashamed of confessing. In the difficult task, then, of diagnosing the disease to which a joint lesion is due, we must be guided not only by its form and locality, but also by a number of general symptoms carefully analysed.

**The Joints in Gout.**—A chronic joint affection is found most often in the great toe, but other frequent seats of trouble are the ankles, knees, wrists, and especially the fingers. In the latter we may find spindle-shaped swellings or the most extraordinary distortions, complete removal of phalanges, and deflexion of the fingers to the ulnar side. A search may reveal tophi in the ears or elsewhere, and crystals of urate of soda may be found in the serum from a blister. The patient is generally a male, over 30, often alcoholic, perhaps a painter or other worker in lead, and he may give an account of a series of acute attacks, the first of which commenced in the great toe. The kidneys are often affected, the heart may be large or in other cases almost normal, and in the acute attacks there is not much fever or sweating, but some glossy redness over a joint, and during convalescence some pitting and peeling of the skin is seen about it. Besides the frequent presence of granular casts in the urine Levison relies a good deal on the radiograph in doubtful cases. This shows the clear streak in the joints remaining intact. The outline of the bones is normal, but with pale areas here and there. Many writers think that rheumatism may occur in gouty subjects, and thus occasionally complicate the lesion.

**Rheumatoid Arthritis or Polyarthritis Deformans.**—The affected joints in the pure form of this disease are very numerous and symmetrical on each side of the body. There are fusiform enlargements of the first phalangeal joints, but from there the affection spreads upwards, and may attack every articulation in the body. Sometimes the two first metacarpo-phalangeal joints on each hand are enlarged before any others, and the wrist deformity may be very marked. Before long, true grating sounds are heard on rubbing the ends of the bones together; a remarkable wasting of the muscles near the damaged joints, patches of pigment on the face, and freckles on the arms, cold or sweating hands, and a rapid pulse are frequent concomitants. The patients are usually women, often young, with a tubercular history, ill-nourished, and complain of a constant pain in the joints, with darting neuralgic pains elsewhere. The fingers are deflected to the ulnar side even more frequently than in gout, and the jaw is attacked in this disease and in gonorrhœal rheumatism alone. Neither the heart, nor the kidneys, nor the temperature are affected as a rule. Garrod notices the value of Heberden's nodes, as often marking an early or abortive attack of this disease, though in later life they may be simply the result of wear and tear. Lastly, the radiograph shows the obliteration of cartilage and the osteophytes at the edge of the bones, which indeed can be seen by the naked eye. Occasionally similar diseases are seen in children, and all are probably of septic origin.

**The Mono-articular Rheumatism of the Aged.**—This usually commences in one hip or shoulder, and may extend to other joints after a time. There is grating, and sometimes dislocation, much wearing pain, and perhaps shortening of the thigh.

**True Rheumatism.**—A mono-articular lesion is sometimes seen following acute rheumatism in younger people, perhaps in the shoulder or knee, and generally yields after awhile to treatment, though a few cases, probably septic ones in reality, end in fibrous anchylosis. Garrod adds to this class the instances of frequently recurring joint attacks which now and then replace or follow ordinary rheumatic fever. We may get an indefinite number of these abortive attacks, like the *petit mal* of epilepsy. Sometimes they are almost continuous for months or years. But in all these manifestations of true rheumatism there is no destruction of cartilage or bony outgrowth, and little beyond pain and stiffness with some creaking to be noted about the joint, which may be transferred after awhile to another articulation. Garrod, in opposition to some writers, thinks that there is little pyrexia or tendency to heart trouble in this chronic adult rheumatism. We are apt to forget, too, that though full doses of salicylates may quickly remove every symptom of acute rheumatism, a tendency to relapse remains for some time. Hence an apparently chronic case may be the last flicker of an acute attack, which neglect may fan into a flame.

**Gonorrhœal Arthritis.**—Some days or months after infection a joint may be attacked, often it is a large one, sometimes the tempero-maxillary. The eyes and the muscles are occasionally affected. There is much pain and effusion, but rarely any heart affection, pyrexia, or shifting from joint to joint, and though most resistant to remedies (indeed, salicylates have no effect here) when once cured it never returns unless a fresh gonorrhœa is contracted. Suppuration occasionally happens, but the great risk is fibrous anchylosis. Many cases of "rheumatism" during the puerperium are probably of this nature.

**Dysenteric and Scarlatinal Arthritis.**—These rarely become chronic. The former may be of the dry type, attacking many joints at once, or accompanied by effusions and fibrous adhesions. The latter is most often of a specific type. This occurs in the early days of the fever, and leaves few traces afterwards. A true rheumatic form is occasionally seen during convalescence, with heart troubles, and perhaps fibrous adhesions.

**Hemophiliac Arthritis.** though rare, should not be forgotten. One form is recurrent, being simply due to hæmorrhage into the joints, so that we have a tender fluctuating joint swelling in a person known to be a bleeder. When these attacks have gone on for years a further change takes place in the joint, akin to that seen in rheumatoid arthritis, and much bony outgrowth takes place with destruction of the cartilages.

**The Arthropathy of Tabes.**—There is great effusion here, and from the extraordinary destruction of ligaments, cartilage, and even bone, the unnatural mobility of the joint is extreme. Occasionally, we see an outgrowth, but usually the head of the bone is simply atrophied. The other signs of tabes, such as the gait, the loss of knee jerks, and the eye-changes may complete the diagnosis.

**Syphilitic Arthritis.**—There is a synovitis seen in the early stages with or without some thickening of the synovial membranes. It commences with little or no pain, and then the swelling intermits, *i.e.*, disappears and reappears at times in a curious way. The tertiary or late secondary forms are marked by more implication of the synovia and firm thickening of the joint, with occasional destruction of cartilage, though the disease usually tends to become stationary at an early stage.

**Tuberculous Joints.**—We can merely refer to this wide group. Here are noticed pain, synovial thickening, with a curious pallor over the joint, wasting of the muscles, and a specially flexed position. Perhaps sinuses and grating are seen in later stages, with violent starting pain. Hip-joint disease, spinal caries, and other special forms have their own symptoms, often definite enough, though at times it may be hard to diagnose one in an early stage from rheumatic or other affections.

